# Hydrophobicity of Benzene-Based Surfactants and Its Effect on Bubble Coalescence Inhibition

**DOI:** 10.3390/molecules29215042

**Published:** 2024-10-25

**Authors:** Rafael Del Río-Arrillaga, Arturo A. García-Figueroa, José L. López-Cervantes, Boris Albijanic, Jesús Gracia-Fadrique

**Affiliations:** 1 Laboratorio de Superficies, Departamento de Fisicoquímica, Facultad de Química, Universidad Nacional Autónoma de México, Ciudad de México 04510, Mexico; rafael.delrioa@gmail.com (R.D.R.-A.); joseluislopezcervantes@gmail.com (J.L.L.-C.); jgraciaf@unam.mx (J.G.-F.); 2 Western Australian School of Mines: Minerals, Energy and Chemical Engineering, Curtin University, Kalgorlie, WA 6430, Australia

**Keywords:** surfactants, bubble coalescence, hydrophobicity, salicylic acid-based compounds

## Abstract

Bubble coalescence plays a critical role in optimizing biological and industrial processes, impacting efficiency in areas such as fermentation, wastewater treatment, and foaming control. While the relationship between chemical structure and bubble coalescence has been thoroughly explored for inorganic ions, limited data exist on organic ions and surfactants, despite their widespread use in these industries. This study addresses this gap by investigating the effects of surfactant hydrophobicity and bubble size on coalescence behavior at a flat air–liquid interface and within a bubble column. Surface tension measurements were employed to assess surfactant hydrophobicity, while bubble size and coalescence time were analyzed to determine their respective influences. The results reveal a novel quantitative relationship between surfactant hydrophobicity and the half-coalescence inhibition concentration (HCIC), a new variable introduced in this study. This relationship demonstrates that as hydrophobicity increases, the HCIC also rises, providing a new relationship between surfactant hydrophobicity and bubble coalescence. While it is well-known that more hydrophobic molecules delay coalescence, this is the first time a direct, proportional relationship has been established with HCIC, offering a new parameter for predicting and controlling coalescence phenomena.

## 1. Introduction

Bubbles play a significant role in various industrial processes, including foam stability, gas separation, and enhanced oil recovery [1,2,3]. The coalescence of bubbles can greatly impact the efficiency and effectiveness of these processes. Understanding the factors that influence bubble coalescence and exploring methods to inhibit it are crucial for optimizing such applications. One critical area where preventing bubble coalescence is particularly important is in decompression sickness [4,5,6]. Decompression sickness occurs when divers ascend rapidly, leading to the formation of nitrogen bubbles in capillary veins, which can block blood flow and potentially result in limb loss. Therefore, inhibiting bubble coalescence is essential in reducing the harmful effects of decompression sickness. Benzene-based surfactants effect on bubbles have been analyzed in relation to decompression sickness [7]. In this study, other benzene-based surfactants, commonly used in industries such as textiles [8], cosmetics [9], and pharmaceuticals [10] were also examined to analyze the effect of different functional groups on bubble coalescence.

Bubble coalescence has been studied in relation to inorganic ions, and a clear connection between ion presence and bubble coalescence has been observed [11,12]. For surfactants, bubble coalescence time increases with surfactant concentration, and this relationship has been found to be governed by surface tension gradients [13,14]. Bubble size has also been shown to correlate with surfactant hydrophobicity, with a notable increase in size observed for nonionic ethoxylated surfactants and an even more pronounced effect for ionic surfactants [15]. This phenomenon is attributed to the strong affinity of highly hydrophobic surfactants for the surface, which leads to increased surface pressure [16]. The rise in surface pressure, resulting from repulsive forces between surfactant molecules at the interface, creates a barrier that prevents adjacent bubbles from coalescing.

Liquid film stability has been linked to surface tension gradients and forces within the liquid films. Sheludko and Vrij proposed that the rupture of liquid films is controlled by the surface compression modulus, or Gibbs elasticity [17], which is also dependent on surface tension gradients. High-surface-tension gradients have been observed in surfactants with highly hydrophobic molecules, such as benzene-based surfactants [18]. Therefore, surface-tension gradients and the overall behavior of surfactants, especially their hydrophobicity [19], which is critical in many surface phenomena [20,21,22], can be determined through surface-tension measurements by fitting-surface equations of state.

In this paper, we investigate the relationship between the hydrophobicity of benzene-based surfactants and bubble coalescence. We measured the surface tension of liquid solutions, bubble size, air-bubble–liquid-surface coalescence, and coalescence inhibition to establish a clearer understanding of how surfactant hydrophobicity affects bubble size, coalescence, and coalescence inhibition.

## 2. Results and Discussion

### 2.1. Coalescence Time

**Figure 1:**
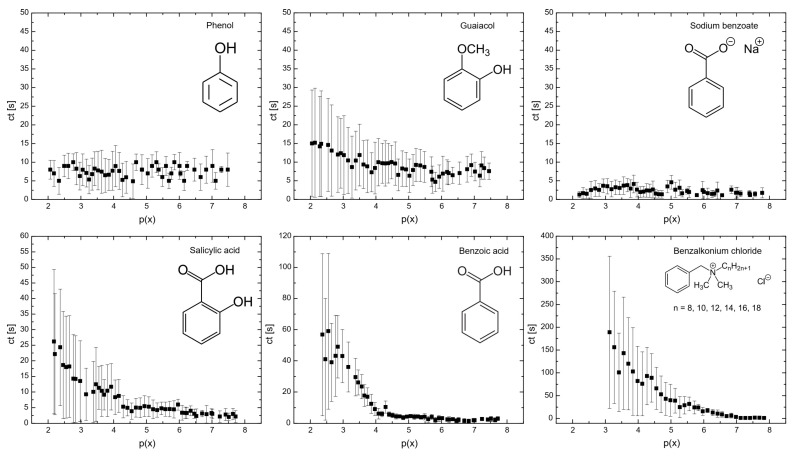
Flat air–liquid coalescence time measured in the bubble coalescence measurement cell as a function of p(x) = −log(x).

Figure 1 illustrates the influence of surfactants on the coalescence time between bubbles. As shown, at a mole fraction of 10^−3^ (p(x) = 3), the coalescence time decreases in the following order: benzalkonium chloride > benzoic acid > salicylic acid > guaiacol > phenol > sodium benzoate. A similar trend was observed for the surfactant concentrations required to achieve a surface tension of 65 mN/m (see Figure 6), with the order: phenol > sodium benzoate > guaiacol > benzoic acid > salicylic acid > benzalkonium chloride. This indicates a strong correlation between coalescence time and surfactant concentration, a relationship that has been previously reported in other studies [23,24]. At higher concentrations of surfactants, the bubble surface becomes saturated with surfactant molecules, forming a barrier and increasing the repulsive forces between bubble interfaces. A further increase in surfactant concentration caused the surfactant molecules to migrate from the unstretched regions to the stretched regions of the bubble surfaces. This migration generated a restoring force that resisted deformation of bubbles, prolonging the coalescence time.

**Figure 2:**
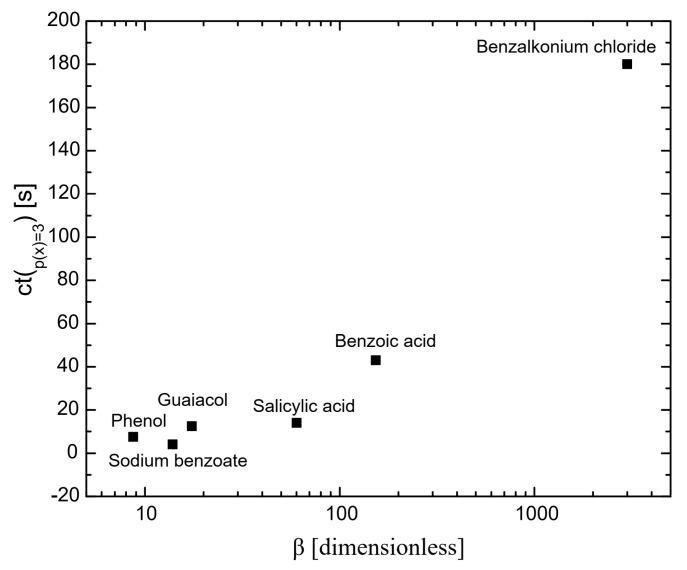
The influence of hydrophobicity on coalescence time; the coalescence time was considered at p(x) and is 3 for all the chemicals.

Figure 2 presents the coalescence time as a function of surfactant hydrophobicity at the maximum measured concentration (p(x) = 3). It shows that the greater the hydrophobicity, the longer the coalescence time. This is because highly hydrophobic surfactants adsorb more effectively on bubble surfaces than less hydrophobic surfactants [25,26]. Highly hydrophobic molecules adsorb onto bubble surfaces, which lowers the solution-free energy and results in greater surface tension gradients than those produced by less hydrophobic molecules. This drives a stronger Marangoni effect, leading highly hydrophobic molecules to flow more efficiently toward thinner regions of the gas–liquid interface, equalizing the surface tension and enhancing interface stability [27]. Consequently, bubble surfaces are more stable with highly hydrophobic surfactants, preventing coalescence. These results are consistent with those reported by Henry and Craig [28] in their studies on sucrose, other sugars, and urea.

### 2.2. Coalescence Inhibition

**Figure 3:**
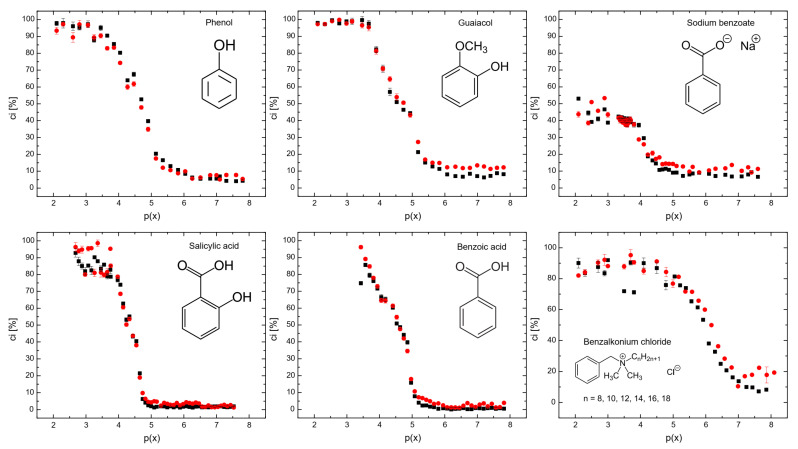
The coalescence inhibition percentage measured in the coalescence measurement column (see Figure 3) as a function of p(x) = −log(x). ▪ Lower detector, ● upper detector.

Figure 3 shows the coalescence inhibition percentage for all the surfactants. At lower concentrations for all the surfactants, the coalescence inhibition percentage can be neglected. The reason is that insufficient surfactants adsorbed onto the bubbles, resulting in weaker repulsive forces between bubbles and allowing coalescence to occur. When the surfactant concentration increases, the surface tension of the bubbles is significantly reduced, generating strong repulsive forces between bubbles. This occurs because surfactant molecules form a dense layer around the bubbles, preventing the coalescence of bubbles.

Figure 3 also shows that sodium benzoate was the least effective in coalescence inhibition. Sodium benzoate showed a low hydrophobicity, as seen in Table 2. Additionally, sodium benzoate solubility of 556 g/L [29] was the highest for all the surfactants used, i.e., the solubility of phenol, guaiacol, salicylic acid, benzoic acid, and benzalkonium chloride are 84 g/L, 17 g/L, 2 g/L, 1.7 g/L, and 544 g/L, respectively [29]. It means that the low hydrophobicity and high solubility of sodium benzoate decreased surfactant adsorption onto bubble surfaces [27]. Benzalkonium chloride and sodium benzoate, despite being surfactants, may be less effective for coalescence inhibition, as shown in Figure 3, at higher surfactant concentrations. It is noteworthy that non-ionic molecules coalescence inhibition seems to start to increase strongly at around p(x) = 5, while ionic molecules do not follow this trend. The reason for this could be due to the surface charge of these surfactants. However, further investigations are needed to better understand this behavior.

**Figure 4:**
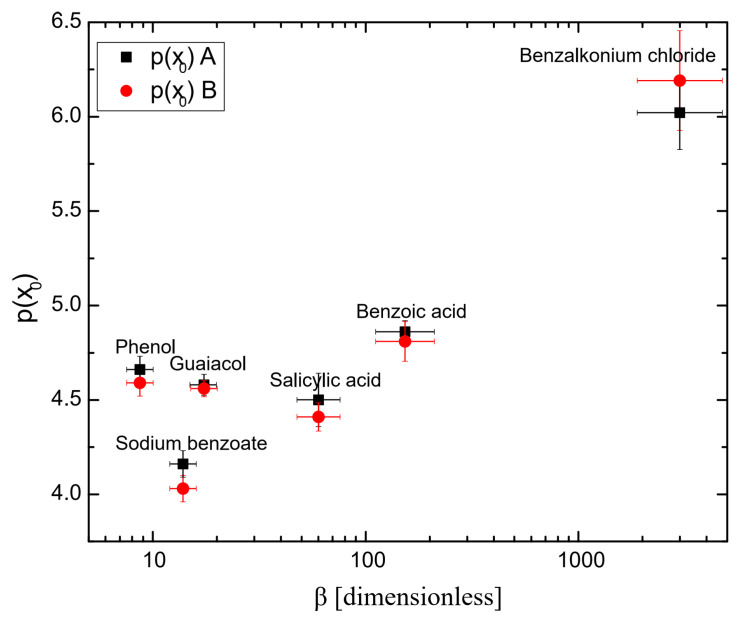
Half coalescence inhibition concentration p(x_0_) as a function of hydrophobicity. A upper detector; B lower detector.

**Figure 6:**
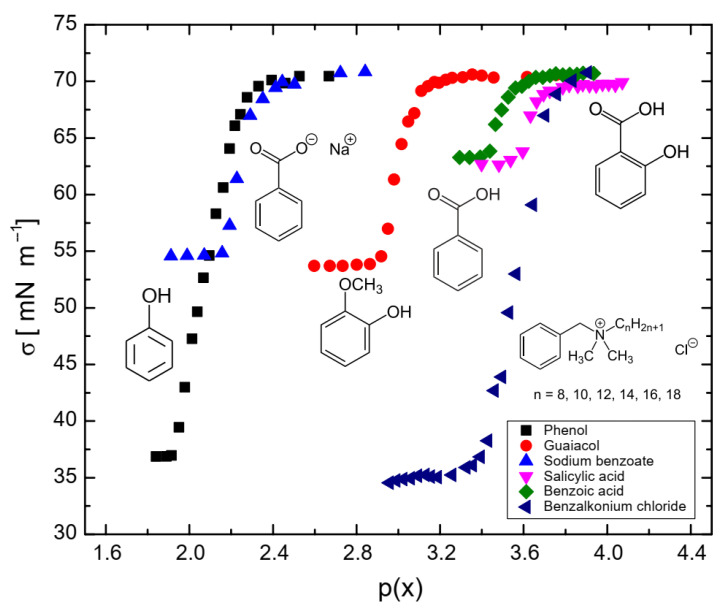
Surface tension of benzene-based surfactants as a function of p(x) = −log(x).

Tables S1 and S2 show the parameters obtained using Equation (3) and the fitting quality for all the surfactants (see the Supplementary Materials). Table 1 shows the parameter p(x_0_) of Equation (3) for all surfactants. p(x_0_) is the concentration required for achieving 50% of the maximum inhibition and thus was considered as the half coalescence inhibition concentration HCIC. Figure 4 shows the half coalescence inhibition concentration p(x_0_) as a function of hydrophobicity β. It is shown that the higher the surfactant hydrophobicity, the higher the values for (x_0_). This is because highly hydrophobic surfactants are more attracted to the nonpolar air phase than to the polar water phase. As a result, they preferentially position themselves at the air–aqueous interface, decreasing their free energy and the surface tension. Consequently, a lower concentration of surfactant molecules is needed to decrease surface tension, as seen in Figure 6. Lastly, a lower concentration of a highly hydrophobic surfactant is needed to achieve the same level of surface activity as a higher concentration of a less hydrophobic surfactant. Phenol and guaiacol are slightly above the trend observed in Figure 4, probably because phenol and guaiacol do not dissociate into ionic species, leading to a lower half coalescence inhibition concentration (i.e., higher p(x)) than expected because nonionic molecules produce a weaker repulsive interaction.

**Table 1:** Half coalescence inhibition concentration p(x_0_).

Surfactant	Upper Detector	Lower Detector
	p(x_0_) ^A^	p(x_0_) ^B^
Phenol	4.66	4.59
Guaiacol	4.58	4.56
Sodium Benzoate	4.16	4.03
Salicylic acid	4.50	4.41
Benzoic acid	4.86	4.81
Benzalkonium chloride	6.02	6.19

^A^ upper detector; ^B^ lower detector.

### 2.3. The Relationship Between Bubble Size and Hydrophobicity

**Figure 5:**
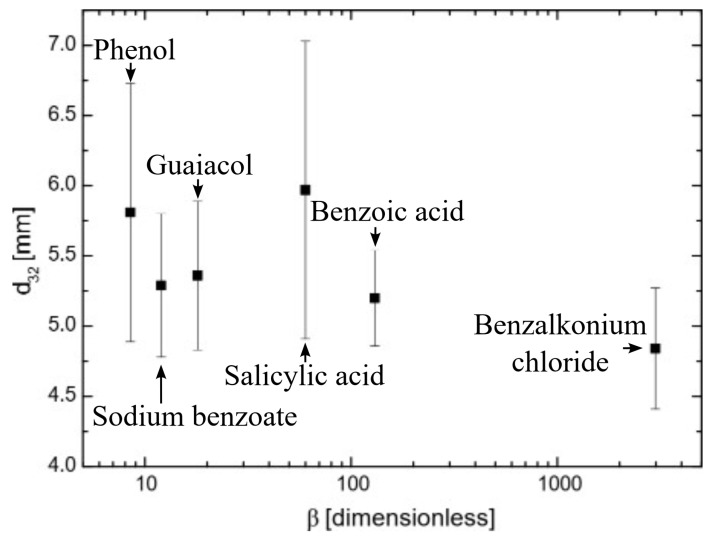
Sauter means diameter of single bubbles as a function of surfactant hydrophobicity.

Figure 5 shows the Sauter mean diameter of surfactant bubbles using the bubble column (see Figure 8) as a function of hydrophobicity at p(x) = 3 for all the surfactants used. As seen in Figure 5, the increase in hydrophobicity slightly affected the bubble size even though the hydrophobicity increased more than 100 times. Bubble size has been found to be highly dependent on flow dynamics, which can be modified by surfactant properties [30]. However, surfactant concentration had a more significant effect on bubble size [31]. Given that all surfactants were at highly inhibiting coalescence concentrations (p(x) = 3), no significant effects were observed on bubble size.

## 3. Materials and Methods

### 3.1. Materials

Surface tension and coalescence measurements were conducted for phenol, guaiacol, sodium benzoate, salicylic acid, benzoic acid, and benzalkonium chloride. All chemicals were purchased from Sigma-Aldrich (CDMX, Mexico), with purities exceeding 99%, except for benzalkonium chloride, which had a purity greater than 95%. Benzalkonium chloride was used as a mixture of derivatives with varying carbon chain lengths, comprising 1.2% octyl derivative, 2.6% decyl derivative, 37.7% dodecyl derivative, 44.5% tetradecyl derivative, 9.7% hexadecyl derivative, and 4.3% octadecyl derivative.

### 3.2. Surface Tension Measurements

The surface tension of aqueous surfactant solutions at various concentrations was measured using a ring method to calculate the hydrophobicity of the surfactants. The methodology for surface-tension measurements and hydrophobicity calculations is described in detail elsewhere [32]. The solutions were placed in a temperature-controlled cell for 2 h prior to measurement to ensure thermal equilibrium. The ring was then slowly raised from the solution surface at a rate of 2 mm/h, the maximum force was recorded, and the ring was returned to the solution. This procedure was repeated for all solutions, with three measurements taken for each, and the experimental error did not exceed 0.1 mN/m.

Surface-tension measurements as a function of surfactant concentration were fitted to a Langmuir surface equation of state (Equation (1)). In this equation, σ_0_ represents the surface tension of water (71.81 mN/m), A is equal to *Γ_s_RT* (where *Γ_s_* is the maximum surface coverage by the surfactant, *R* is the universal gas constant, *T* is the temperature), and *β* represents the hydrophobicity. The molecular parameters derived from the Langmuir surface equation of state are shown in Table 2. The concentration *p*(*x*) is expressed as the negative logarithm of the mole fraction *x*, as indicated in Equation (2).

As shown in Figure 6, the concentration of surfactants required to achieve a surface tension of 65 mN/m followed the order: phenol > sodium benzoate > guaiacol > benzoic acid > salicylic acid > benzalkonium chloride. Hydrophobicity (i.e., *β*) was higher when a carboxyl group was attached to the aromatic ring and lower when hydroxyl or methoxy groups were present. The surface maximum coverage (*Γ_s_*) was similar across all surfactants due to their molecular similarities, even for benzalkonium chloride, as the alkyl chains would orient themselves perpendicularly to the surface at saturation, resulting in a similar surface area. This is consistent with data reported for other surfactants in the literature [33,34].

(1) $$\sigma_{0}-\sigma =\Gamma_{s}RT\ln\left(1+\beta x\right)$$

(2) $$p\left(x\right)=-log(x)$$

**Table 2:** Molecular parameters obtained by Equation (1) fitting to surface tension measurements.

Surfactant	β	*Γ_s_RT* [mmol/m^2^]
Phenol	7.8 ± 3.2	17 ± 2.6
Guaiacol	11.3 ± 1.5	16 ± 3.7
Sodium benzoate	19.4 ± 4.1	15 ± 1.8
Salicylic acid	55.5 ± 10.6	17 ± 2.2
Benzoic acid	125.9 ± 31.8	10 ± 1.3
Benzalkonium chloride	3001 ± 365.9	20 ± 1.7

### 3.3. Coalescence Measurements

#### 3.3.1. Coalescence Time Measurements

**Figure 7:**
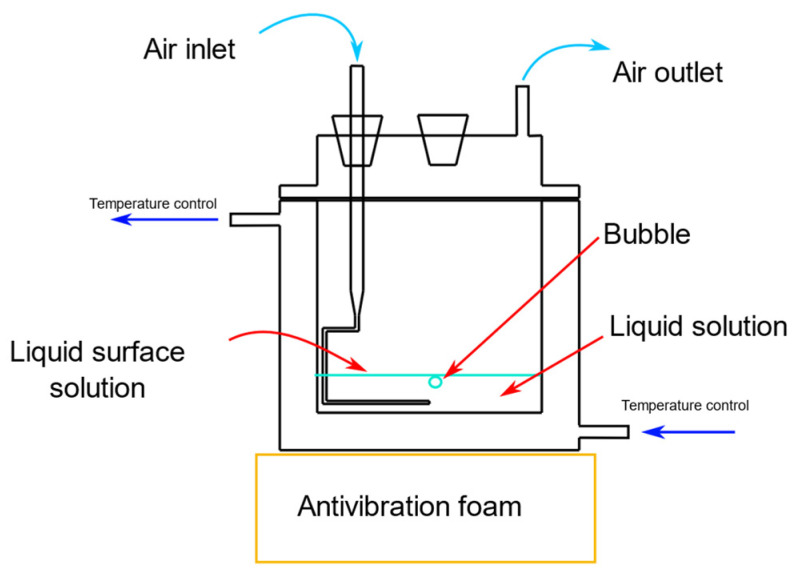
Bubble flat air–liquid coalescence measurement cell.

Coalescence time measurements were conducted in a temperature-controlled cell, as shown in Figure 7. A modified glass pipette was used to generate the bubbles, with air pumped at a rate of 1 mL/min using a Cole Parmer^®^ syringe pump (CDMX, Mexico). Different surfactant solutions (phenol, sodium benzoate, guaiacol, benzoic acid, salicylic acid, or benzalkonium chloride) were introduced into the cell. When a bubble detached from the pipette tip and reached the liquid surface, the coalescence time (*ct*), defined as the time from when the bubble reached the surface to the moment it burst, was recorded [35,36]. For each concentration, 30 bubbles were measured to ensure accurate results.

#### 3.3.2. Coalescence Inhibition Measurements

**Figure 8:**
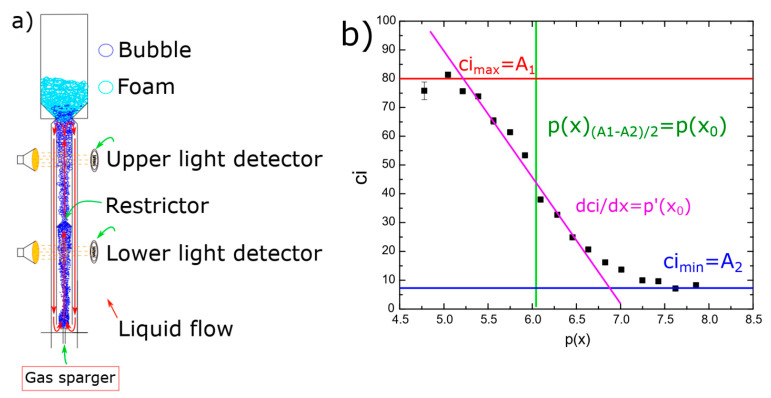
Coalescence measurements: (**a**) coalescence column, (**b**) coalescence inhibition percentage as a function of surfactant concentration fitted to Equation (3); experimental data are of benzalkonium chloride.

Coalescence measurements [28,37] were conducted using a square bubble column with a height of 60 cm and a cross-sectional area of 9 cm^2^. Inside the column, a smaller tube (height: 56.5 cm, cross-sectional area: 6 cm^2^) was placed to facilitate the recirculation of liquids and bubbles (see Figure 8). Air was introduced at an airflow rate of 0.8 LPM through a fritted-glass sparger (0.3 ± 0.1 mm pore size) located at the bottom of the bubble column. The column was filled with 200 mL of each surfactant solutions (phenol, sodium benzoate, guaiacol, benzoic acid, salicylic acid, or benzalkonium chloride).

The bubble column was divided into two sections by a restrictor: the first section had a height of 40 cm, and the second section had a height of 20 cm. Measurements were taken once a constant foam height of 10 cm was achieved and after 5 min of steady-state conditions. Two light detectors were positioned at different heights to measure light dispersion caused by passing air bubbles (see Figure 8). Higher light dispersion indicated a greater concentration of bubbles, which corresponded to increased coalescence inhibition. Two standards were used to calibrate the measurements: water, representing 0% coalescence inhibition, and a critical micelle concentration of sodium dodecyl sulfate (8 mmol/L), representing 100% coalescence inhibition.

The coalescence inhibition measurements produced a sigmoidal curve, as shown in Figure 8b. The data were fitted to the new model that relates coalescence inhibition to surfactant concentration. Equation (3) was used to determine the coalescence inhibition (*ci*) for the various surfactant solutions based on the experimental results.

(3) $$ci=A_{2}+\frac{A_{1}-A_{2}}{1+e^{\frac{p\left(x\right)-p(x_{0})}{p^{\prime }(x_{0})}}}$$

where *A*_1_ and *A*_2_ are the maximum and minimum inhibition percentage of a surfactant, respectively; *p*(*x*) and *p*(*x*_0_) are the logarithmic concentration of the surfactant, as given in Equation (2); *x* is the molar fractions of the surfactant in a solution; *x*_0_ represents the molar fractions of the surfactant in a solution when the coalescence inhibition is 50%; therefore, *p*(*x*_0_) is the half-coalescence inhibition concentration (HCIC); and finally, *p′*(*x*_0_) is the slope at HCIC.

### 3.4. Bubble Size Measurements

Bubble size measurements were taken with a camera (Nikon D3100 with an AF-S Nikkor 18–55 mm lens, CDMX, Mexico) in a dark room with a light source behind the column or cell. A total of 30 bubbles were measured in the bubble flat air–liquid coalescence measurement cell, and 100 bubbles were measured in the coalescence column. The Sauter mean diameter of bubbles of the flat air coalescence measurement cell is shown in Table 3.

## 4. Conclusions

Previous studies have examined the relationship between bubble coalescence and the ionic charge of inorganic ions, but the connection between bubble coalescence and the hydrophobicity of surfactants has not been explored in depth. This investigation aimed to establish a link between bubble coalescence and the molecular structure of surfactants, specifically focusing on hydrophobicity. Our results revealed a linear relationship between surfactant hydrophobicity and coalescence time. Additionally, a clear relationship between surfactant concentration and bubble coalescence inhibition was identified. A critical concentration was determined for all surfactants, beyond which coalescence inhibition increased rapidly.

This study illuminates the complex relationship between surfactant hydrophobicity, concentration, and bubble coalescence dynamics, providing a foundation for potential practical applications in various fields, including the prevention of decompression sickness.

## Figures and Tables

**Table 3:** Sauter mean diameter of bubbles of the flat air coalescence measurement cell (see Figure 7).

Surfactant	d_32_ [mm]
Phenol	0.49 ± 0.06
Salicylic acid	0.65 ± 0.12
Benzalkonium chloride	0.73 ± 0.23
Guaiacol	0.88 ± 0.19
Benzoic acid	1.17 ± 0.32
Sodium benzoate	1.87 ± 0.31

## Data Availability

Data are contained within the article and Supplementary Materials.

## Supplementary Materials

The following supporting information can be downloaded at: https://www.mdpi.com/article/10.3390/molecules29215042/s1, Table S1. Equation (3) fitting variables values. Table S2. Fitting quality for Equation (3).
