# Supplementary material for: Hydrophobicity of Benzene-Based Surfactants and Its Effect on Bubble Coalescence Inhibition
Source: Molecules. 2024 Oct 25;29(21):5042. doi: 10.3390/molecules29215042 (PMC11547379; doi:10.3390/molecules29215042)
Supplement: Supplementary file 1 [file molecules-29-05042-s001.zip › molecules-3249914-supplementary.pdf]

Supplementary material

Table S1 shows the parameters obtained using Equation 3, meaning of all parameters is shown in Figure 3. Table S2 shows the fitting quality for Equation 3.

Table S1. Equation 3 fitting variables values.

Surfactant	Upper detector				Lower detector			
	$A_1{}^A$	$A_2{}^A$	$p(x_0)^A$	$p'(x_0)^A$	$A_1{}^B$	$A_2{}^B$	$p(x_0)^B$	$p'(x_0)^B$
Phenol	93.42	5.71	4.66	0.35	91.65	6.25	4.59	0.34
Guaiacol	100	6.04	4.58	0.42	98.80	10.95	4.56	0.38
Sodium Benzoate	40.64	10.13	4.16	0.14	41.91	14.16	4.03	0.19
Salicylic acid	77.27	0	4.50	0.17	84.82	0	4.41	0.21
Benzoic acid	60.13	1.02	4.86	0.12	63.89	4.03	4.81	0.14
Benzalkonium chloride	85.62	7.43	6.02	0.38	77.53	16.77	6.19	0.23

A upper detector, B lower detector

Table S2. Fitting quality for Equation 3.

	Upper detector		Lower detector	
	R^2	MPE (%)	R^2	MPE (%)
Phenol	0.9881	8±5	0.9908	10±8
Guaiacol	0.9685	10±7	0.9825	8±5
Sodium benzoate	0.9906	5±4	0.9811	4±4
Salicylic acid	0.9735	44±58	0.9695	23±22
Benzoic acid	0.9793	25±42	0.9923	10±7
Benzalkonium chloride	0.9972	5±5	0.9657	11±18

MPE is the mean error (%).
